# The feasibility and benefit of unsupervised at-home training of minimally invasive surgical skills

**DOI:** 10.1007/s00464-022-09424-2

**Published:** 2022-07-28

**Authors:** Maja Joosten, Vera Hillemans, Guus M. J. Bökkerink, Ivo de Blaauw, Bas H. Verhoeven, Sanne M. B. I. Botden

**Affiliations:** 1 Department of Pediatric Surgery, Radboudumc - Amalia Children’s Hospital, Nijmegen, The Netherlands; 2 Princess Máxima Center for Pediatric Oncology, Utrecht, The Netherlands; 3 Department of Surgery, Radboudumc, Nijmegen, The Netherlands; 4 Department of Surgery, Zuyderland, Heerlen, The Netherlands; 5 Department of Pediatric Surgery, Radboud University Medical Center, Geert Grooteplein Zuid 10 route 618, 6500 HB Nijmegen, The Netherlands

**Keywords:** MIS, Simulation training, At-home training, Unsupervised, Skill acquisition

## Abstract

**Background:**

Simulation-based training may be used to acquire MIS skills. While mostly done in a simulation center, it is proposed that this training can be undertaken at-home as well. The aim of this study is to evaluate whether unsupervised at-home training and assessment of MIS skills is feasible and results in increased MIS skills.

**Methods:**

Medical doctors and senior medical students were tested on their innate abilities by performing a pre-test on a take-home simulator. Henceforth, they followed a two-week interval training practicing two advanced MIS skills (an interrupted suture with knot tying task and a precise peg transfer task) and subsequently performed a post-test. Both tests and all training moments were performed at home. Performance was measured using motion analysis software (SurgTrac) and by expert-assessment and self-assessment using a competency assessment tool for MIS suturing (LS-CAT).

**Results:**

A total of 38 participants enrolled in the study. Participants improved significantly between the pre-test and the post-test for both tasks. They were faster (632 s vs. 213 s, *p* < 0.001) and more efficient (distance of instrument tips: 9.8 m vs. 3.4 m, *p* = 0.001) in the suturing task. Total LS-CAT scores, rated by an expert, improved significantly with a decrease from 36 at the pre-test to 20 at the post-test (*p* < 0.001) and showed a strong correlation with self-assessment scores (*R* 0.771, *p* < 0.001).

The precise peg transfer task was completed faster (300 s vs. 163 s, *p* < 0.001) and more efficient as well (14.8 m vs. 5.7 m, *p* = 0.005). Additionally, they placed more rings correctly (7 vs. 12, *p* = 0.010).

**Conclusion:**

Unsupervised at-home training and assessment of MIS skills is feasible and resulted in an evident increase in skills. Especially in times of less exposure in the clinical setting and less education on training locations this can aid in improving MIS skills.

Teaching residents in the operating room is effective but may be inefficient and costly [1, 2]. An alternative is training preclinically using simulation models. Simulation-based training for minimally invasive surgery (MIS) skills has proven to be effective and efficient in developing transferable skills [3–5]. However, these studies used expensive high-fidelity MIS equipment and training was based in simulation centers, making it costly and not readily available. An alternative is using low-fidelity simulators. This has previously been proven effective in the development of surgical skills as well [6–8]. The true benefit of low-fidelity, low-budget simulators is best appreciated when the simulator can be used at-home [9]. Firstly, at-home training has the advantage that it can be done when convenient and avoids trainees from having to train when feeling fatigued and stressed [9]. Trainees can follow a practice schedule that is more optimal for skill development and retention, such as interval training or spaced learning [24]. In contrast, training in simulation centers is often based on the concept of bulk training or massed practice with little repetition. At-home training allows for distribution of practice and the repetition needed for deliberate practice [9].

The second advantage of at-home training is that it is very cost effective, because there is no need for costly simulation centers [9]. Especially when using low-budget box trainers, at-home training comes with little cost [10].

Lastly, during periods of little clinical exposure or hands-on courses (such as during the COVID-19 pandemic) it may be used by trainees to acquire and retain surgical skills needed for complex MIS procedures [11–13].

An important aspect of at-home training is that it is unsupervised and trainees do not receive immediate expert feedback. Especially when training complex procedural skills receiving feedback is important [14]. However, this does not necessarily have to be expert feedback. Previous studies have shown promising results with self-assessment and self-rating, which can help guide trainees during unsupervised training at home [15].

The aim of this study is to evaluate the feasibility and benefit of unsupervised at-home training and assessment of MIS skills.

## Methods

### Participants

Senior medical students, medical doctors, and PhD students of Radboudumc, Nijmegen, and UMC Utrecht, Utrecht, the Netherlands were recruited to participate in this study in the period of May–September 2020. Participants with surgical knowledge and interest but without (unsupervised/independent) surgical experience were included. All participants completed an informed consent form and agreed with anonymous processing of the data.

Approval of the ethics committee of the institution Radboudumc was obtained and approval of the ethical board approval of the ethics committee of Arnhem and Nijmegen was waived. The study was performed in accordance with the ethical standards as laid down in the 1964 Declaration of Helsinki and its later amendments or comparable ethical standards.

### MIS at-home simulator

**Fig. 1:**
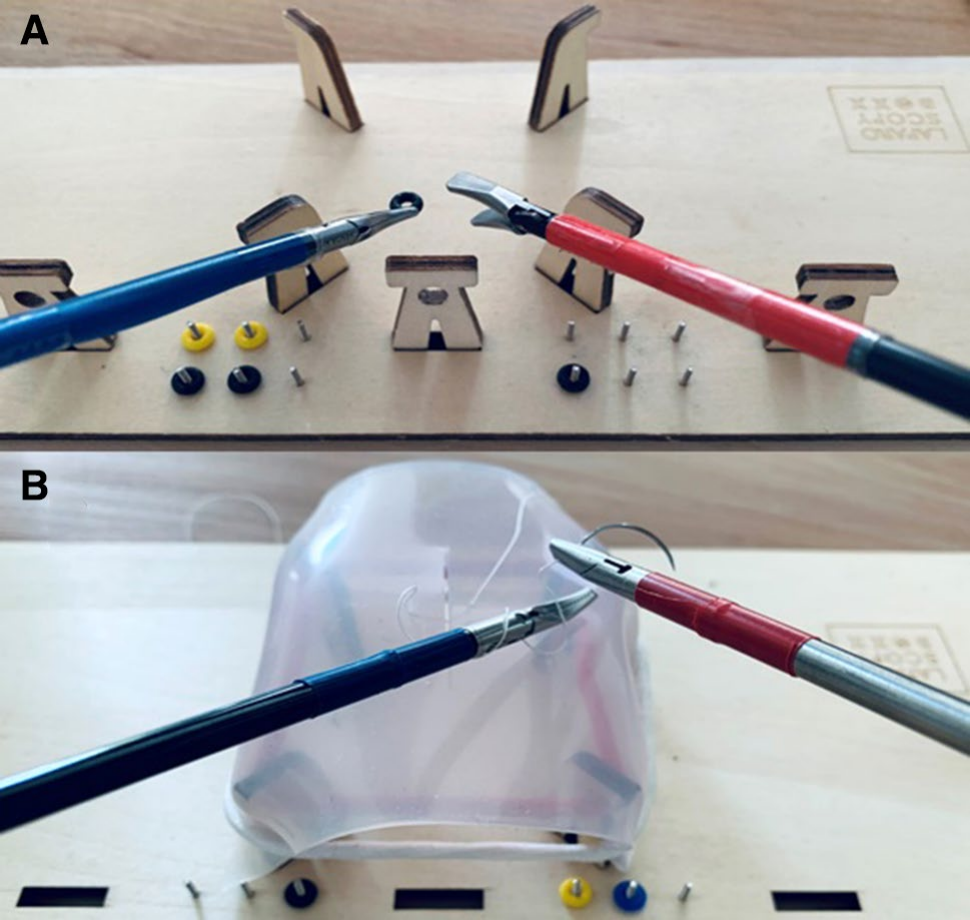
**a** Precise peg transfer task. **b** Suturing task performed on the LaparoscopyBoxx take-home simulator [16, 22]

The take-home simulator used in this study was the LaparoscopyBoxx (Outside the Boxx, Nijmegen, The Netherlands) [10, 16]. This is a low cost wooden box trainer, which is distributed as a build-yourself package (Fig. 1). This MIS take-home trainer is light weight and easily transported. It has three or five instrument ports and has an opening in the center of the top panel, which is designed for the camera of a smartphone or a tablet. In this study a tablet (Lenovo P10) was used to serve as the screen. The instruments used during this study were a 5 mm needle holder, two graspers (one curved and one straight), a dissector and scissors.

### Tasks

All participants practiced a precise peg transfer task and a suturing task (both adapted from Fundamentals of Laparoscopic Surgery) [17]. Peg transfer was chosen because it is one of the more basic tasks, allowing trainees to learn basic MIS skills. Suturing and knot tying on the other hand is one of the most advanced FLS tasks, requiring more dexterity and precision which results in better discrimination between skilled and unskilled trainees.

The precise peg transfer task required participants to move the six small pegs, which were placed on pins on the left side of the board, to the right side of the board, and subsequently back to the left side (Fig. 1a). Pegs had to be lifted with a grasper in the non-dominant hand and transferred mid-air to the dominant hand. Following, each peg was placed on a pin on the right side of the board. After correct placement of all pegs, the task was repeated to the other side. The maximum time for this task was five minutes.

The suturing task consisted of placing one interrupted suture in a suturing pad (15 cm braided 3–0 suture with 17 mm 3/8 curved needle) and performing an intracorporeal surgical knot (Fig. 1b), without a time limit.

All training and test sessions were performed at-home, without a supervisor present. During training sessions written instructions, a poster with the steps and video instructions on the separate tasks were provided for guidance. No other supervision or guidance was given. During the test moments both the precise peg transfer task and suturing task were performed.

### Evaluation of skills

#### Motion analysis

For motion analysis the SurgTrac software (Eosurgical ltd., Edinburgh, Scotland, United Kingdom) was used [18, 19]. The application was installed on the tablet and used during training (Lenovo P10) and test sessions to track the instrument tips. This software allows for instrument tracking by means of the colored markings on the instrument tips (red for the instrument in the dominant hand and blue for the instrument in the non-dominant hand). The parameters provided by this software system for each task were time needed to perform the procedure (seconds), mean off-screen time for the instruments of the right and left hand (percentage), distance traveled by the instrument tips (meters), workspace in between the instrument tips (meters), handedness (percentage), speed (m/s), acceleration (m/s^2^), and smoothness (m/s^3^). SurgTrac was used to assess the performance of both the precise peg transfer task and the suturing task.

#### Expert-assessment and self-assessment

**Fig. 2:**
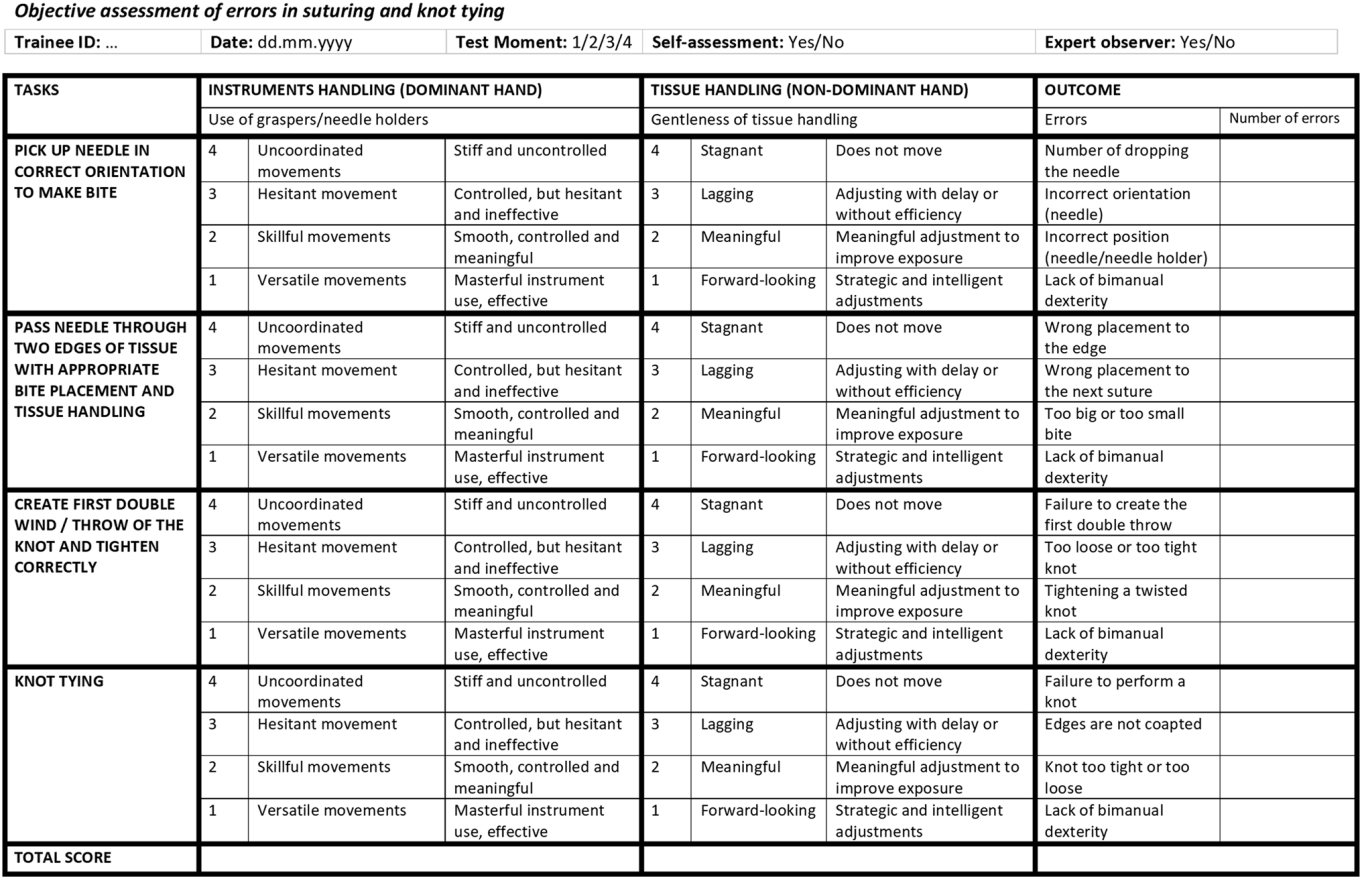
Competency assessment tool for laparoscopic suturing (LS-CAT) [20]

The competency assessment tool for laparoscopic suturing (LS-CAT) was used for self-assessment and expert-assessment of the suturing task. This is a previously developed and validated competency assessment tool [20]. It consists of two vertical columns (one for instrument handling and one for tissue handling) and four horizontal rows which represent the four different tasks of laparoscopic suturing (pick-up needle in correct orientation to make bite; pass needle through two edges of tissue with appropriate bite placement and tissue handling; create first double throw of the knot and tighten correctly; knot tying). This results in eight separate items which are scored on a scale of one to four. A lower score indicates a more proficient performance with a score of eight as a perfect score and 32 as a poor performance score. A third column represents the number of errors which are scored on four domains for each task, resulting in 16 separate items (Fig. 2). The LS-CAT was completed by participants after the pre-test and the post-test as self-assessment. Additionally, a blinded expert observer assessed the video performance of all test sessions of the participants and scored their performance on the LS-CAT for expert-assessment.

### Outcome parameters

Primary outcome parameters for the precise peg transfer were total time needed and distance traveled by the instrument tips. Secondary outcome parameters were the number of correctly transferred pegs and errors (number of pegs dropped) which were noted by blinded researchers, after evaluation of each test video.

The performance of the suturing task during the test sessions was assessed by the parameters of the SurgTrac software. Increase in skill level (primary outcome) was measured by the total time needed and distance traveled by the instrument tips, because previous studies have shown evident construct validity for these two parameters [21, 22]. Furthermore, the LS-CAT was used for self-assessment and expert-assessment to provide an objective assessment of the quality of the suture and the knot (secondary outcome). All test videos were assessed by a blinded expert observer, who had extensive experience using the LS-CAT form.

### Protocol

All participants completed a short questionnaire on their demographic data and surgical experience. They performed a pre-test to determine their innate abilities. Thereafter, they performed an at-home interval training schedule in which they practiced six times within two weeks. Written and video instruction of the two surgical tasks were provided as well as a self-assessment form of the suturing task. Practice sessions were 90 min and both tasks were practiced each session, all sessions were logged in SurgTrac. This training schedule was based on previous research showing superiority of interval training over bulk training [23]. Participants did not receive further guidance during training or test sessions due to the COVID-19 pandemic. After the training program, all participants performed a post-test. The test sessions were recorded with the camera of the tablet used by the participants and videos were submitted to the researchers. Videos of the test sessions were assessed on errors for the ring transfer task and scored with the LS-CAT for the suturing task by a blinded researcher.

### Statistical analysis

Data were analyzed using IBM SPSS statistics 25 (Armonk, NY: IBM Corp) and R (3.6.2, rmcorr package). Differences in performance between the two time points were analyzed with a related samples Wilcoxon. A *p*-value of < 0.05 was considered statistically significant.

The correlation between self- and expert-assessment of the LS-CAT was determined using the Spearman’s rho (repeated measures correlation for the combination of time points), on a 2-tailed significance level of *p* < 0.05. An *R* of < 0.200 was considered a very weak or no correlation, an *R* of 0.200–0.399 was considered a weak correlation, an *R* of 0.400–0.599 a moderate correlation, an *R* of 0.600–0.799 a strong correlation, and an *R* of ≥ 0.800 was considered a very strong correlation.

A sample size calculation was performed with a power of 0.80 and an alpha of 0.05. In order to find a difference of 360 s in total time needed for the suturing task, 34 participants were needed. To account for possible drop-out during the study, 38 participants were included.

## Results

A total of 38 participants enrolled in the study. Mean time between the pre-test and the post- test was 15 days (SD 1.4). The majority of participants were female (60%) and the average age was 25 years (SD 2.2). The majority were medical students (66%), followed by medical doctors not in training to become surgeons (26%) and PhD-candidates (8%).

The majority of participants had seen open (97%) and MIS (97%) surgical procedures and assisted in open (89%) or MIS (82%) procedures. No participants had performed any MIS procedures by themselves (Table 1).

**Table 1:** Demographics properties of the participants

Total group (*n* = 38)			
Age	25.0 (2.2)		
Gender (%)			
Male	15 (40)		
Female	23 (60)		
Profession (%)			
Medical student	25 (66)		
Medical doctor	10 (26)		
PhD-candidate	3 (8.0)		

	Performed	Assisted	Seen
Open surgery			
0	29 (76)	4 (11)	0 (0)
0–5	6 (17)	5 (13)	2 (5.3)
5–10	2 (5.3)	6 (16)	2 (5.3)
10–20	1 (2.6)	9 (24)	6 (16)
20–50	0 (0)	9 (24)	13 (34)
> 50	0 (0)	5 (13)	15 (40)
Minimally invasive surgery			
0	38 (100)	7 (18)	0 (0)
0–5	0 (0)	18 (47)	3 (7.9)
5–10	0 (0)	13 (34)	2 (5.3)
10–20	0 (0)	0 (0)	16 (42)
20–50	0 (0)	0 (0)	10 (26)
> 50	0 (0)	0 (0)	7 (18)

Values are depicted as mean with standard deviation or number with percentage

### Skill acquisition

#### Peg transfer

Participants improved significantly between the pre-test and post-test for both tasks. For the precise peg transfer task, the time needed to complete the task decreased evidently from the maximum set limit (300 s) to 163 s (*p* < 0.001). The total number of correctly placed pegs almost doubled (7 pegs vs. 12 pegs, *p* = 0.010), whereas the number of pegs dropped decreased from 1 to 0 pegs (*p* = 0.010). The distance of the instrument tips decreased considerably (14.8 m vs. 5.7 m, *p* = 0.005). The smoothness increased from 0.08 to 0.18 mm/s^3^ (*p* < 0.001) (Table 2).

**Table 2:** SurgTrac parameters of the precise peg transfer task

Peg transfer task	Pre-test	Post-test	p-value
SurgTrac parameters			
Total time (s)	300 (3.0)	163 (63)	< 0.001
Distance (m)	14.8 (30)	5.7 (9.9)	0.005
Pegs transferred			
Total pegs (N)	7.0 (8.0)	12 (0.0)	0.010
Pegs dropped (N)	1.0 (2.0)	0.0 (1.0)	0.010

Values are stated as median with interquartile range. Groups are compared with a related samples Wilcoxon

#### Suturing

The total time needed for the suturing task decreased significantly (632 vs. 213 s, *p* < 0.001). The distance traveled of the instrument tips decreased by more than half (9.8 vs. 3.4 m, *p* = 0.001) (Table 3,).

**Table 3:** SurgTrac parameters of the suturing task and expert-assessment with LS-CAT

Suturing task	Pre-test	Post-test	*p*-value
SurgTrac parameters			
Total time (s)	632 (411)	213 (119)	< 0.001
Distance (m)	9.8 (13)	3.4 (5.0)	0.001
LS-CAT (expert-assessment)			
Instrument handling	15 (1.0)	9.0 (6.0)	< 0.001
Tissue handling	16 (2.0)	8.0 (6.0)	< 0.001
Errors	6.0 (5.0)	1.0 (2.0)	< 0.001
Total score	37 (7.0)	19 (12)	< 0.001

Values are stated as median with interquartile range. Groups are compared with a related samples Wilcoxon

Expert assessment of videos of the suturing task showed an evident increase in skills between the pre-test and the post-test. The expert total LS-CAT scores decreased from 36 at the pre-test to 20 at the post-test (*p* < 0.001) (Table 1). Instrument handling scores decreased by one third (pre-test: 15 vs post-test: 9.4, *p* < 0.001) with the most improvement for the task ‘create first double wind/throw of the knot and tighten correctly’ (*p* < 0.001). A similar increase in skills was found for tissue handling as well (pre-test: 15 vs. post-test: 9.5, *p* < 0.001). The total number of errors decreased evidently from 6.6 at the pre-test to 1.3 at the post-test (*p* < 0.001) (Table 3).

### Self-assessment suturing task

The self-assessment scores (how participants rated their own performance; a lower score reflecting a better performance) on the LS-CAT decreased significantly between the pre-test and post-test (total score 45 to 26 (*p* < 0.001) (Table 4). Instrument handling decreased from 14 to 10 (*p* < 0.001) with the most improvement in the score for ‘knot tying’ (from 4.0 to 2.5, *p* < 0.001). Tissue handling decreased from 13 to 10 (*p* < 0.001), with a decrease from 3.0 to 2.0 for each of the four steps (*p* < 0.001 for all).

**Table 4:** Correlation between expert-assessment and self-assessment calculated with the Spearman’s rho (< 0.200 no correlation; 0.200–0.399 weak correlation; 0.400–0.599 moderate correlation; 0.600–0.799 strong correlation, ≥ 0.800 very strong correlation)

Correlation LS-CAT scores	Expert-assessment	Self-assessment	Spearman’s rho	*p*-value
Instrument handling	14 (3.9)	12 (2.6)	0.815 (0.63–0.92)	< 0.001
Tissue handling	14 (3.9)	12 (2.5)	0.815 (0.62–0.92)	< 0.001
Errors	3.0 (3.9)	11 (12)	0.680 (0.39–0.85)	< 0.001
Total score	29 (11)	34 (15)	0.771 (0.54–0.89)	< 0.001

Scores are depicted as median with interquartile range. Rho is depicted with 95% confidence intervalss

The number of self-reported errors decreased significantly as well (from 16 to 6, *p* < 0.001), with the most evident decrease for the steps ‘pick up the needle and make the first bite’ (from 7.0 to 2.0, *p* < 0.001) and ‘knot tying’ (from 3.0 to 1.0, *p* < 0.001).

#### Correlation expert- and self-assessment

**Fig. 3:**
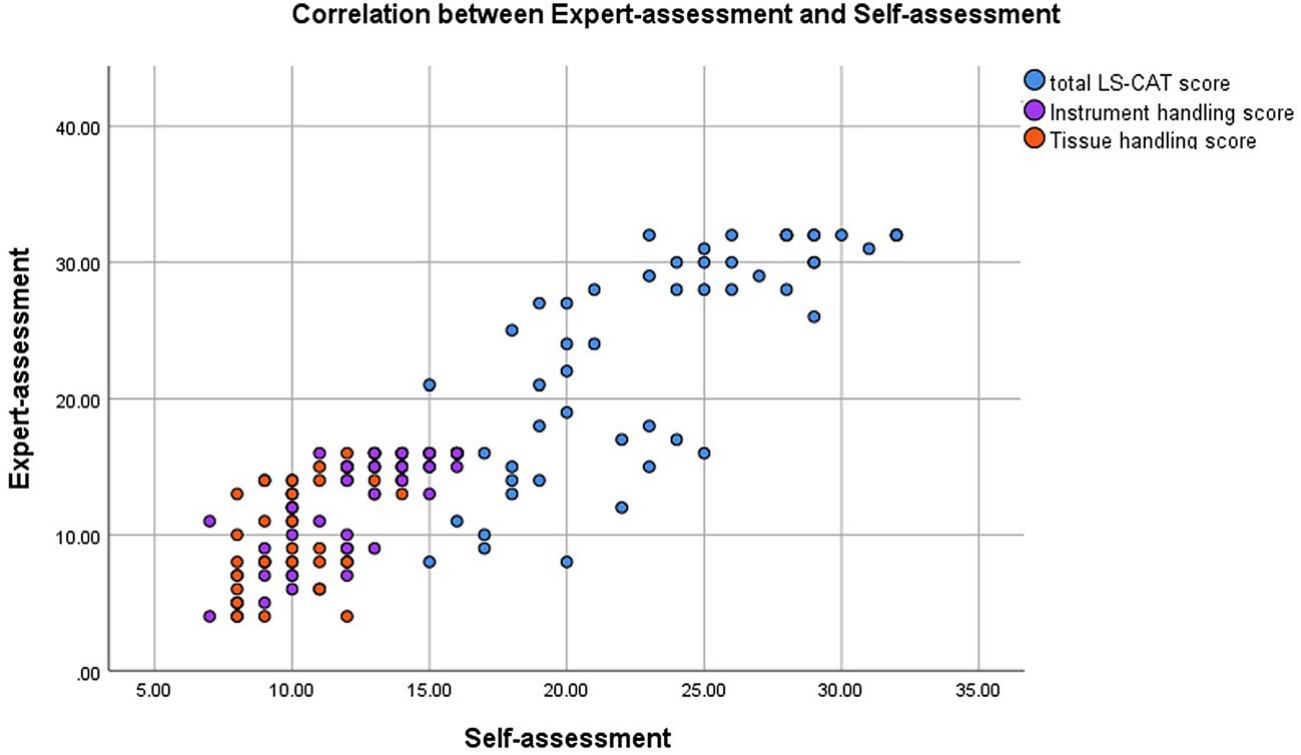
Scatterplot of the correlation between Expert-assessment and self-assessment for the total LS-CAT scores and the components instrument handling and tissue handling

Comparison of self-assessment and expert-assessment scores showed a strong correlation for the total LS-CAT scores (*R* 0.771, *p* < 0.001) and for the errors (*R* 0.680, *p* < 0.001). Additionally, a very strong correlation was found for the components instrument handling (*R* 0.815, *p* < 0.001) and tissue handling (*R* 0.815, *p* < 0.001) (Table 4 and Fig. 3). As shown in Table 4, the correlations were weaker when evaluated for the separate test moments.

## Discussion

This study showed an evident increase in skills between the pre-test and the post-test indicating that unsupervised at-home training of MIS skills is feasible and beneficial. This study is the first in measuring skill increase after unsupervised at-home training by expert-assessment, self-assessment, and movement analysis parameters. All three outcome measures indicate an increase in skills after a period of unsupervised at-home training. This implies that at-home training without expert guidance is beneficial for MIS skill acquisition.

Written instructions, posters with the steps of the tasks and videos of the tasks were provided, the participants received no expert guidance or feedback during training or test sessions. Nevertheless, there was strong evidence for an increase in the skills of the participants. This finding is in line with previous research of Bruwaene et al., who compared a group with expert feedback with an unsupervised training group. In this study no difference in performance of MIS suturing scores were detected after an initial supervised training session [24]. Korndorffer et al. found similar results showing that at-home unsupervised training resulted in excellent basic MIS skill acquisition [9]. Our study shows that unsupervised at-home training is feasible even without initial supervised training.

The trainees in this study used self-assessment to evaluate their test sessions. Previous research has shown that self-assessment and reflection-before-practice results in an increase in technical skills [25, 26]. Evaluation before and after practice enables trainees to identify tasks or parts of procedures where improvements can be made. Our study shows a strong correlation between self-assessment and expert-assessment scores, however, only a weak correlation was found when the pre-test and post-test were analyzed separately. This might be due to smaller sample size when time points are analyzed separately. Furthermore, there were large differences in the number of errors scored by participants versus the number of errors scored by experts for the pre-test, resulting in a larger difference in total scores for that specific time point. Another explanation might be the use of trainees that were unskilled for MIS suturing, or that the trainees did not evaluate the assessment form before the start of the first training and were not aware what was expected for a good performance. Previous research has shown that trainees that are unskilled in a particular domain lack the ability to realize that they are incompetent (unconsciously incompetent) [25–30]. As a result, unskilled trainees will be less able to recognize competence compared to more competent peers or experts. With increased experience of the trainees, the correlation between self- and expert-assessment will increase, which was seen in our results as well (pre-test *R* 0.071 vs post-test *R* 0.299). Self-assessment can aid in increasing competence by making trainees aware of their strengths and weaknesses. Although it is no replacement for expert-assessment it may be beneficial for guidance and evaluation during unsupervised at-home training [25].

At-home training has many advantages. It enables trainees to train during optimal moments for themselves and account for fatigue or negative stressors, which could affect their performance. This type of effortful and deliberate practice leads to explicit motor learning [31]. Self-regulated practice sessions have been shown to have a positive effect on motor learning retention [32, 33]. Furthermore, at-home trainees can devote their full attention to learning the MIS task, whereas training in a simulation center or hospital setting may cause distractions and limited focus [9, 34]. Moreover, at-home training using box trainers is less costly than using a simulation center [35] and is better suited for spaced learning. The latter resulting in increased skill acquisition and retention. However, merely providing surgical residents with box trainers for at-home training does not seem to incentivise trainees to practice [36, 37]. A systematic review by Gostlow et al. on motivation for participation in simulation-based training of MIS found lack of available time to be the greatest barrier. Furthermore, trainees favored training for specific procedures over basic tasks [38]. These motivators need to be taken into account when developing an at-home training schedule for trainees.

### Limitations

Although surgical residents are the target group of this study, they were not included as participants. Residents are exposed to more confounders (such as training and workshops on MIS skills and exposure to MIS procedures in the clinical setting) and subsequently introduce bias in the results. Therefore, participants with surgical knowledge and interest, but without MIS experience, were included.

Because test and training sessions were conducted at-home, the setting in which they took place may have varied among participants (e.g., background noise, fatigue). Participants were instructed to practice in a setting and at a time that they perceived as convenient. Variation in settings are therefore inevitable and could not be controlled by the researchers. However, this also poses one of the main advantages of at-home training: it may be done at any place and time that is convenient.

Unsupervised training may tempt participants in deviating from protocol, however, all sessions were logged with SurgTrac and all test sessions were recorded and no deviations from protocol were encountered.

## Conclusion

Unsupervised at-home training of MIS skills is feasible and leads to an evident increase in MIS skills. Self-assessment may be beneficial for guidance and evaluation during unsupervised at-home training. This is especially important in times of less exposure in the clinical setting and limited opportunities for education on location or expert guidance.

## References

[1] Bridges M, Diamond DL (1999). The financial impact of teaching surgical residents in the operating room. Am J Surg.

[2] Hawasli A, Featherstone R, Lloyd L, Vorhees M (1996). Laparoscopic training in residency program. J Laparoendosc Surg.

[3] Scott DJ, Bergen PC, Rege RV, Laycock R, Tesfay ST, Valentine RJ, Euhus DM, Jeyarajah DR, Thompson WM, Jones DB (2000). Laparoscopic training on bench models: better and more cost effective than operating room experience?. J Am Coll Surg.

[4] Seymour NE, Gallagher AG, Roman SA, O'Brien MK, Bansal VK, Andersen DK, Satava RM (2002). Virtual reality training improves operating room performance: results of a randomized, double-blinded study. Ann Surg.

[5] Ahlberg G, Enochsson L, Gallagher AG, Hedman L, Hogman C, McClusky DA, Ramel S, Smith CD, Arvidsson D (2007). Proficiency-based virtual reality training significantly reduces the error rate for residents during their first 10 laparoscopic cholecystectomies. Am J Surg.

[6] Bruynzeel H, Bruin AFJ, Bonjer HJ, Lange JF, Hop WCJ, Ayodeji ID, Kazemier G (2007). Desktop simulator: key to universal training?. Surg Endosc.

[7] Dayan AB, Ziv A, Berkenstadt H, Munz Y (2008). A simple, low-cost platform for basic laparoscopic skills training. Surg Innov.

[8] Wanzel KR, Matsumoto ED, Hamstra SJ, Anastakis HD (2002). Teaching technical skills: training on a simple, inexpensive, and portable model. Plast Reconstr Surg.

[9] Korndorffer JR, Bellows CF, Tekian A, Harris IB, Downing SM (2012). Effective home laparoscopic simulation training: a preliminary evaluation of an improved training paradigm. Am J Surg.

[10] Bökkerink GMJ, Joosten M, Leijte E, Verhoeven BH, De Blaauw I, Botden SMBI (2021). Take-home laparoscopy simulators in pediatric surgery: is more expensive better?. J Laparoendosc & Adv Surg Tech.

[11] Dedeilia A, Sotiropoulos MG, Hanrahan JG, Janga D, Dedeilias P, Sideris M (2020). Medical and surgical education challenges and innovations in the COVID-19 era: a systematic review. In Vivo.

[12] Potts JR (2020). Residency and fellowship program accreditation: effects of the novel coronavirus (COVID-19) pandemic. J Am Coll Surg.

[13] Nassar AH, Zern NK, McIntyre LK, Lynge D, Smith CA, Petersen RP, Horvath KD, Wood DE (2020). Emergency restructuring of a general surgery residency program during the coronavirus disease 2019 pandemic: the university of Washington experience. JAMA Surg.

[14] Strandbygaard J, Bjerrum F, Maagaard M, Winkel P, Larsen CR, Ringsted C, Gluud C, Grantcharov T, Ottesen B, Sorensen JL (2013). Instructor feedback versus no instructor feedback on performance in a laparoscopic virtual reality simulator: a randomized trial. Ann Surg.

[15] Scott DJ, Ritter EM, Tesfay ST, Pimentel EA, Nagji A, Fried GM (2008). Certification pass rate of 100 % for fundamentals of laparoscopic surgery skills after proficiency-based training. Surg Endosc.

[16] LaparoscopyBoxx, accessed 13 Jan, 2022

[17] Zendejas B, Ruparel RK, Cook DA (2016). Validity evidence for the Fundamentals of laparoscopic surgery (FLS) program as an assessment tool: a systematic review. Surg Endosc.

[18] SurgTrac software, eoSurgical, accessed 13 Jan, 2022

[19] Partridge RW, Hughes MA, Brennan PM, Hennessey,  (2014). Accessible laparoscopic instrument tracking (“InsTrac”): construct validity in a take-home box simulator. J Laparoendosc Adv Surg Tech.

[20] IJgosse WM, Leijte E, Ganni S, Luursema J, Francis NK, Jkimowicz JJ, Botden SMBI,  (2020). Competency assessment tool for laparoscopic suturing: development and reliability evaluation. Surg Endosc.

[21] Leijte E, Arts E, Witteman B, Jakimowicz JJ, De Blaauw I, Botden SMBI (2019). Construct, content and face validity of the eoSim laparoscopic simulator on advanced suturing tasks. Surg Endosc.

[22] Mansoor SM, Våpenstad C, Mårvik R, Glomsaker T, Bliksøen M (2020). Construct validity of eoSim—a low-cost and portable laparoscopic simulator. Minim Invasive Ther Allied Technol.

[23] Joosten M, Bökkerink GMJ, Stals JJM, Leijte E, De Blaauw I, Botden SMBI (2021). The Effect of an interval training on skill retention of high-complex low-volume minimal invasive pediatric surgery skills: a pilot study. J Laparoendosc Adv Surg Tech.

[24] Van Bruwaene S, Schijven MP, Miserez M (2013). Maintenance training for laparoscopic suturing: the quest for the perfect timing and training model: a randomized trial. Surg Endosc.

[25] Joosten M, Bökkerink GMJ, Verhoeven BH, Sutcliffe J, de Blaauw I, Botden SMBI (2021). Are self-assessment and peer assessment of added value in training complex pediatric surgical skills?. Eur J Pediatr Surg.

[26] Ganni S, Botden SMBI, Schaap DP, Verhoeven BH, Goossens RHM, Jakimowicz JJ (2018). "Reflection-before-practice" improves self-assessment and end-performance in laparoscopic surgical skills training. J Surg Educ.

[27] Evans AW, Leeson RMA, Petrie A (2007). Reliability of peer and self-assessment scores compared with trainers' scores following third molar surgery. Med Educ.

[28] Evans AW, Leeson RM, Newton-John TR, Petrie A (2005). The influence of self-deception and impression management upon self-assessment in oral surgery. Br Dent J.

[29] Kruger J, Dunning D (1999). Unskilled and unaware of it: how difficulties in recognizing one's own incompetence lead to inflated self-assessments. J Pers Soc Psychol.

[30] Boud D (1995). Enhancing learning through self-assessment.

[31] Poolton JM, Masters RS, Maxwell JP (2007). Passing thoughts on the evolutionary stability of implicit motor behaviour: performance retention under physiological fatigue. Conscious Cogn.

[32] Brydges R, Carnahan H, Safir O, Dubrowski A (2009). How effective is self-guided learning of clinical technical skills?: It's all about process. Med Educ.

[33] Keetch KM, Lee TD (2007). The effect of self-regulated and experimenter imposed practice schedule on motor learning for tasks of varying difficulty. Res Q Exerc Sport.

[34] Wilson E, Janssens S, McLindon LA, Hewett DG, Jolly B, Beckmann M (2019). Improved laparoscopic skills in gynaecology trainees following a simulation-training program using take-home box trainers. Aust N Z J Obstet Gynaecol.

[35] Franklin BR, Placek SB, Wagner MD, Haviland SM, O'Donnell MT, Ritter EM (2017). Cost comparison of fundamentals of laparoscopic surgery training completed with standard fundamentals of laparoscopic surgery equipment versus low-cost equipment. J Surg Educ.

[36] Nicol LG, Walker KG, Cleland J, Partridge R, Moug SJ (2016). Incentivising practice with take-home laparoscopic simulators in two UK core surgical training programmes. BMJ Simul Technol Enhanc Learn.

[37] Blackhall VI, Cleland J, Wilson P, Moug S, Walker K (2019). Barriers and facilitators to deliberate practice using take-home laparoscopic simulators. Surg Endosc.

[38] Gostlow H, Marlow N, Babidge W, Maddern G (2017). Systematic review of voluntary participation in simulation based laparoscopic skills training: motivators and barriers for surgical trainee attendance. J Surg Ed.

